# Application of Gauss Mutation Genetic Algorithm to Optimize Neural Network in Image Painting Art Teaching

**DOI:** 10.1155/2021/3302617

**Published:** 2021-11-16

**Authors:** Weiming Xing, Jian Zhang, Quan Zou, Jun Lin

**Affiliations:** ^1^Henan Vocational College of Water Conservancy and Environment, Zhengzhou 450008, Henan Province, China; ^2^Zhengzhou University of Industry Technology, School of Art and Design, Zhengzhou 451100, Henan Province, China

## Abstract

With the continuous application of the art industry in various fields, more and more people choose to systematically learn the knowledge of the art industry. In the art major, image painting is one of the important contents of the art major. How to improve students' aesthetic quality and comprehensive professional quality is studied, in which the content learning of image painting art is the key. Therefore, we have carried out technical exploration and result analysis based on Gaussian mutation genetic algorithm to optimize the application of neural network in image painting art teaching. We use Gaussian mutation genetic algorithm to study the neural network optimized teaching cloud platform technology. Compared with the traditional algorithm, the algorithm proposed in this paper has more funny computational efficiency, being able to comprehensively evaluate and improve students' aesthetic quality and comprehensive professional quality. Gaussian mutation genetic algorithm can effectively improve the knowledge search ability of the platform and the running speed of the teaching platform. In the future research in the field of art industry, neural network will optimize the teaching cloud platform technology, which has laid a solid foundation for improving students' aesthetic quality and comprehensive professional quality.

## 1. Introduction

In the face of diversified social development, most of the people's needs for a better life have been met [1]. More and more people begin to pursue the discovery of beauty. Talents in the art industry can also help various fields develop forward and keep up with the changes of the times [2, 3]. Image painting education technology is the main part of the whole art teaching activities. The whole learning time of image painting accounts for more than 50% of all art teaching [4]. How to make students' professional knowledge more stable and creative is a problem worthy of attention in painting art teaching. At present, the problems existing in the teaching of image painting art mainly include the imperfect teaching system and the relatively small functional coverage [5]. The mutual penetration of various professional courses has a great impact on painting teaching. There are great differences in students' overall ability and requirements. Image painting art is a subject with high requirements for its own creativity and thinking ability [6]. More importantly, in teaching, we adhere to the traditional offline platform teaching. It cannot achieve more scientific and intelligent teaching methods [7, 8].

With the development of artificial intelligence and the leading of computer industry, the training ability of machine learning has changed. Neural network algorithm and deep learning are improved and optimized on the original basis [9, 10]. Genetic algorithm (GA) is a deep learning search algorithm that follows natural rules and rules [11]. Its basic principle is the algorithm generated by combining artificial intelligence functions with natural rules [12]. By optimizing the parameters involved, the functional evolution of various neural network algorithms is realized. The main feature of the operation is to query and exchange data information between variables and parameters by retrieval [13, 14]. Genetic algorithm is suitable for solving nonlinear problems or neural network optimization problems. The first simulation test is based on the traditional neural network algorithm. In terms of prediction accuracy and speed, the performance of genetic algorithm is better than that of [15]. Gaussian genetic algorithm has many models. Gaussian mutation is the mutation of search and retrieval function by improving genetic algorithm, that is, the improvement of local search ability, such as search range and direction [16]. Gaussian mutation is also an optimization algorithm. At first, few people paid attention to it, and its scope of use was very small. It is found that the algorithm can improve the prediction system and process complex signal data [17]. The original invention aims to meet the purpose of mass production of finished products. Then, it is gradually used for image model test and sample acquisition.

To improve students' aesthetic quality and comprehensive professional quality, the content learning of image painting art is the key. In order to optimize the application of neural network in image painting art teaching, this paper makes a technical exploration and result analysis based on Gaussian mutation genetic algorithm. This paper creatively uses Gaussian mutation genetic algorithm to study the neural network optimization teaching cloud platform technology. Through a large number of data analysis, the online platform teaching environment can be simulated. It also analyzes the teaching quality evaluation of image painting art.

This paper is divided into three parts. The first part analyzes and summarizes the development trend and current situation of genetic algorithm optimizing neural network and puts forward the technology of Gaussian mutation genetic algorithm optimizing image neural network. The second part is the research on the application technology of Gaussian mutation genetic algorithm optimization neural network in image painting art teaching. This paper mainly analyzes the optimization process of Gaussian mutation genetic algorithm and the support of image rendering teaching cloud platform technology. The local search ability of Gaussian mutation genetic algorithm and the teaching application of MOPSO model are studied. The third part analyzes the application of Gaussian mutation genetic algorithm in image painting art teaching. Firstly, the application effect of painting art teaching cloud platform based on Gaussian mutation genetic algorithm and neural network technology is analyzed. Finally, the application effect of Gaussian mutation genetic algorithm in image painting art teaching is analyzed.

## 2. Related Works

In the process of image painting art creation, we need to get inspiration from the background of the whole society and our own experience. How to integrate the concept of painting into teaching requires not only the support of art major, but also the support of modern science and technology [18]. With the development of the information age, the application of deep learning and neural network algorithm in education and teaching is becoming closer and closer. Using this new algorithm to build a painting art teaching platform can promote the real-time interaction between students and the teaching process [19]. Compared with other neural network algorithms, genetic algorithm is used to optimize neural network. The main characteristics of genetic algorithm optimization are as follows: firstly, genetic algorithm can search data information from multiple data points, which is different from other algorithms starting from a single starting point. It can improve the local solution of the maximum problem [20]. Secondly, genetic algorithm optimizes and visualizes the simulation according to the evaluation information of variable parameter function. It has good global ergodic ability and can obtain the optimal solution in complex data structures [21]. Finally, the neural network optimized by genetic algorithm can process various data together and search the parameters of population variables at the same time, which provides flexibility for the establishment of the model [22].

Some researches simulate and establish models according to natural laws and biological characteristics [23]. Create an algorithm that follows the natural law and is suitable for data acquisition and search traversal function in complex environment. It is also an automatic adaptive optimization probability algorithm. Genetic algorithm includes crossover and mutation operators, which can change the traditional neural network algorithm mode according to its own characteristics. Then, according to the deep learning and genetic algorithm, the machine learning training model is constructed, and the function of genetic algorithm machine learning is realized.

In the 1960s, some scientists began to study natural bionic technology. Based on genetic algorithm, they carry out data processing and model prediction for complex problems [24]. Then, the genetic algorithm is used to optimize various neural networks and applied in the field of computer. At present, this kind of machine learning is used in the medical field, military field, and so on.

The optimized algorithm is promoted to integer operation, floating-point operation, and so on. Various kinds of researchers have effectively applied genetic algorithm optimization neural network to fuzzy calculation of variable parameters and construction of model prediction system [25]. Then, according to the international conference, many deep learning neural network experts explore the advantages of genetic arithmetic optimization neural network.

In the development of deep learning neural network algorithm, firstly, in the research of machine learning training model, neural network algorithm is used to optimize and improve the model. The optimized model is accurate and feasible. It is widely used in pattern recognition, language recognition, artificial intelligence, education and teaching environment, and other fields. The scientists also proposed mutation probability based on the parallel advantage of heredity. According to Gauss variability and genetic algorithm for optimization, the mutation operator is used to improve local search ability. The development of neural network genetic algorithm and the basic principle of Gauss mutation are introduced. To sum up, this paper proposes to use Gauss mutation genetic algorithm to optimize neural network to study its application in painting art teaching. The teaching cloud platform and Gauss mutation optimization mathematical model are introduced in detail.

## 3. Application of Gauss Mutation Genetic Algorithm to Optimize Neural Network in Image Painting Art Teaching

### 3.1. Application of Neural Network Optimized by Genetic Algorithm in Cloud Platform Teaching of Image Painting Art

**Figure 1:**
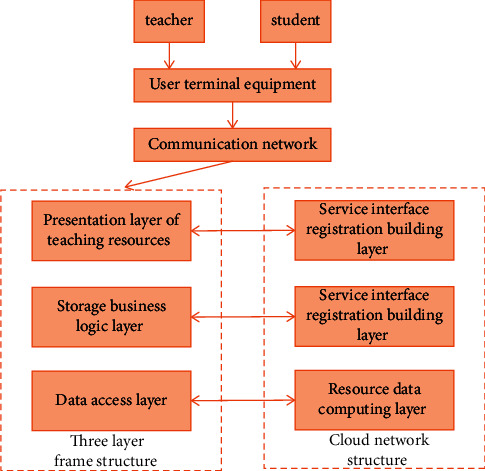
Structural design of cloud platform for image painting art teaching.

In order to improve the quality of painting teaching, we pay attention to the feasibility, effectiveness, and accuracy of teaching. In order to improve the communication and teaching ability of painting teaching, we use a cloud platform different from the traditional teaching environment to support the teaching process. The neural network is optimized according to genetic algorithm, and the scheduling algorithm of the whole platform is improved. From the perspective of data feedback and user response, the optimized neural network is more efficient than the traditional neural network. The establishment of the platform can automatically generate various services and functional software in the cloud environment. Learners do not need to install the cloud service platform on their own computers. They can realize online classes, homework review, homework submission, and other functions through the cloud service platform. This capability can make the user end become a convenient carrier such as mobile phone or tablet computer. The cloud platform can also store the resource information and teaching knowledge after big data search in the public use module and automatically classify and store the data information through the gap and category between different modules. Therefore, the overall server of the cloud platform has high response speed and response speed. The overall structure design of painting art teaching cloud platform is shown in Figure 1.

The structure design of cloud platform mainly includes multiple network framework levels, and the network framework is composed of multiple parts. The specific contents are as follows: teaching platform users, information and communication network, three-tier framework platform, and cloud network structure. The main function of the teaching platform client is to support the whole platform to run intelligent devices and connect to the cloud server and then input or submit the captured data learning information to the cloud. Display the whole process of image painting art teaching through intelligent devices. Information communication is a network of data processing and interaction. The three-tier framework network structure is mainly the presentation structure layer, business logic layer, and data access layer. Its specific function is to respond to the request instructions sent by students and the dynamic instructions between the manager and the platform. Finally, the cloud computing network structure is mainly responsible for students' requests in the process of image painting art teaching, such as scheduling related knowledge content, literature, and picture data. The cloud computing network structure can also manage a large amount of knowledge data in the platform.

In order to improve the running speed and overall efficiency of cloud platform servers. We use genetic algorithm to optimize neural network to improve the teaching platform. Resource equivalence of selection function and priority resource constraint mechanism are added to genetic infrastructure. By analyzing the encoding and decoding rules of variable parameters and combined with the functions of the cloud platform, the encoding method based on resource knowledge is selected. The total number of tasks is calculated as follows:

(1) $$\begin{array}{c} \text{SumTaskNum}=\sum_{t=1}^{J}\text{TaskNum}\left(t\right). \end{array}$$

The calculation formula of the algorithm is as follows:

(2) $$\begin{array}{c} m=\sum_{k=1}^{j}\text{taskNum}\left(k\right)+j. \end{array}$$

Finally, the output data is decoded, and the calculation matrix is designed to obtain the completion time and total amount of each task. The time calculation formula is as follows:

(3) $$\begin{array}{c} \text{JobTime}\left(t\right)=\overset{\text{TaskNum}\left(t\right)}{\underset{i=1}{\max}}\sum_{j=1}^{q}\text{TaskTime}\left(j,i\right). \end{array}$$

The task calculation result data is automatically classified to the coordinates of each calculation data node. The task calculates the completion time on the node variable parameters. The total time is calculated as follows:

(4) $$\begin{array}{c} \text{TotleTime}=\overset{N}{\underset{j=1}{\max}}\sum_{i=1}^{p}\text{NodeTime}\left(j,i\right). \end{array}$$

After defining the relevant parameters of the initial variable population, the corresponding number of processing nodes is matched. Neural network algorithm is used to classify the constrained platform resources. The corresponding variable parameters are produced randomly, and the number of parameters is the number of defined variables. The fitness function in genetic algorithm can capture the advantages of variable parameters and select the next generation evolution direction through the advantages. Finally, the global optimal solution is obtained. The function formula of variable parameter completion time is as follows:

(5) $$\begin{array}{c} f_{1}\left(i\right)=\frac{k\left(i\right)}{J},\;1\leq i\leq S. \end{array}$$

The total time task fitness function is as follows:

(6) $$\begin{array}{c} f_{2}\left(i\right)=\frac{1}{CT\left(i\right)},\;1\leq i\leq S. \end{array}$$

The optimization process of genetic algorithm includes selection, crossover, and mutation, which is the main way to give the next iteration variable data. Firstly, the variable parameters with the highest efficiency are selected, and then the formula is combined with mutation crossover. Calculate the probability of obtaining the whole data:

(7) $$\begin{array}{c} P_{1}\left(i\right)=\frac{f_{1}\left(i\right)}{\sum_{j=1}^{s}f_{1}\left(j\right)},\\ P_{2}\left(i\right)=\frac{f_{2}\left(i\right)}{\sum_{j=1}^{s}f_{2}\left(j\right)}. \end{array}$$

A new search path is generated by mutation crossover operation:

(8) $$\begin{array}{c} P_{c}=\left\{\begin{array}{cc} \frac{k_{1}\left(f_{\max}-f\prime \right)}{f_{\max}-f_{\text{avg}}}, & f\prime \geq f_{\text{avg}},\\ k_{2}, & f\prime <f_{\text{avg}}, \end{array}\right)\\ P_{m}=\left\{\begin{array}{cc} \frac{k_{3}\left(f_{\max}-f\right)}{f_{\max}-f_{\text{avg}}}, & f\geq f_{\text{avg}},\\ k_{4}, & f<f_{\text{avg}}. \end{array}\right) \end{array}$$

Then, the whole platform model is established. Entropy method is used to normalize the data. Firstly, the data source is standardized. The calculation formula is as follows:

(9) $$\begin{array}{c} x_{ij}^{\prime }=\frac{x_{ij}-\bar{x}}{8_{j}\left(2\right)}. \end{array}$$

After standardization, the logarithm requirement is made according to the average value and difference value. The value after translation is

(10) $$\begin{array}{c} Z_{ij}=x_{ij}^{\prime }+A\left(3\right). \end{array}$$

In the formula, the translation value variable and the movement distance are defined. Then, it defines the evaluation index of the image painting art teaching platform:

(11) $$\begin{array}{c} P_{ij}=\frac{Z_{ij}}{\sum_{i=1}^{15}Z_{ij}},\;i=1,2,\ldots ,15;\;j=1,2,\ldots ,82. \end{array}$$

The entropy of each index is calculated as follows:

(12) $$\begin{array}{c} E_{j}=-k\sum_{i=1}^{15}p_{ij}\text{In}\left(p_{ij}\right). \end{array}$$

The entropy index difference coefficient and normalized weight value are as follows:

(13) $$\begin{array}{c} G_{j}=1-E_{j},\\ w_{j}=\frac{G_{j}}{\sum_{j=1}^{82}G_{j}}. \end{array}$$

Finally, the quality calculation formula of the whole data sample in the image painting art teaching resources is as follows:

(14) $$\begin{array}{c} F_{i}=\sum_{j=1}^{m}w_{j}p_{ij}. \end{array}$$

**Figure 2:**
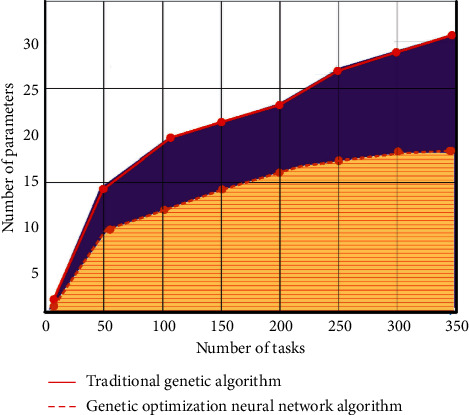
Performance comparison between traditional genetic algorithm and genetic algorithm for neural network optimization.

The specific steps are as follows: firstly, the knowledge resources are coded uniformly to generate the original variable parameter group. The limited resources are classified according to the calculation formula, and the randomly generated variable parameters are used as the initial variables to meet the limited conditions. According to the coding and decoding function, the total number of tasks and function fitness are calculated. Then, the data probability is calculated, and the optimal adaptive parameters are obtained by probability selection. The new iterative individuals are obtained by cross mutation operation of variable parameters. Continue the operation until the global optimal solution is obtained. We will carry out the simulation design experiment on the teaching cloud platform and compare the performance of traditional genetic algorithm and genetic algorithm optimization neural network. The two parameters are set according to the number of processing tasks, and the proportion of the whole task is shown in Figure 2.

**Figure 3:**
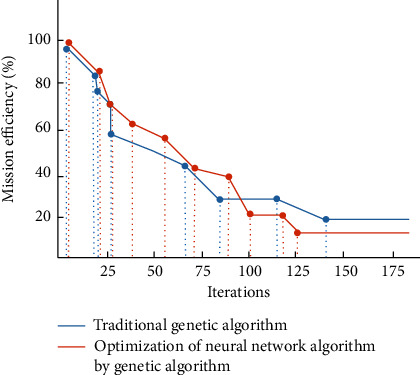
Analysis chart of task completion efficiency and genetic iteration number of the platform.

You can see from the picture, that, compared with the traditional genetic algorithm, the optimized genetic neural network algorithm can update in different number of tasks. The analysis of task completion efficiency and genetic iteration number of the whole platform is shown in Figure 3.

There is a certain gap between the traditional genetic algorithm and the optimized algorithm. The neural network optimized by genetic algorithm can improve the efficiency of task completion and can better deal with complex data problems. In the combination of algorithm and cloud platform, it can optimize the overall performance of the platform and achieve user satisfaction.

### 3.2. Research on Image Painting Art Teaching Technology Based on Gauss Mutation Optimization Search Algorithm and MOPSO Model

**Figure 4:**
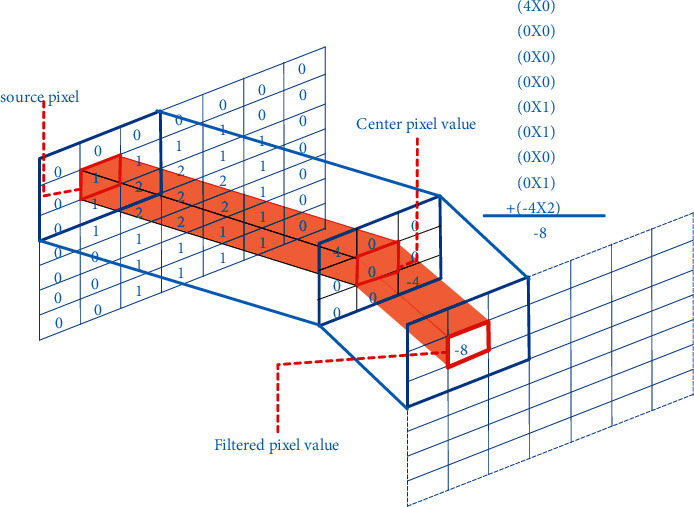
Two-dimensional image pixel matrix structure.

It is necessary to optimize the algorithm of data calculation in the establishment of image painting art teaching simulation model. In the process of data iteration, it is necessary to balance the search range of the algorithm and use random flow patterns to form new variables. However, the defect of this algorithm lies in the poor ability of range retrieval and the slow speed of feedback retrieval. The accuracy of the final data is not high. At present, many scholars have introduced neural network algorithm to optimize the local search ability, which can improve the search performance and feedback speed. In order to increase some operations to improve convergence and diversity, the selection problem of model MOPSO, i.e., mutation operator, is added, mainly to solve the problem that PSO converges to local optimization quickly. Adding disturbance can make it converge to global optimization. Inspired by the above algorithm, this paper proposes a Gauss mutation optimization based search algorithm and a mathematical model MOPSO to calculate the accuracy and feedback speed of the image painting art teaching model. The main method is to update according to the conclusion of the previous algorithm optimization and randomly select variables for local search to generate the latest parameter variables. In this way, the development ability of the algorithm is improved. Finally, Gaussian mutation algorithm is used to update the parameters, so as to improve the feedback speed and accuracy of the whole model. In data acquisition, the image information in the teaching of image painting art is captured first. The structure of the formed two-dimensional image pixel matrix is shown in Figure 4.

**Figure 5:**
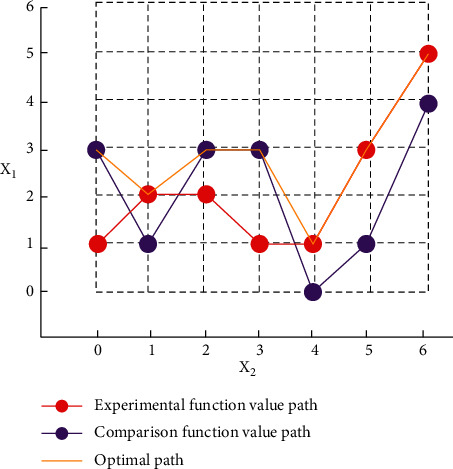
Comparison path chart for solving random distribution of two-dimensional search variables.

In order to improve the accuracy and limit the premature regression, Gaussian mutation algorithm is used to optimize and update the parameter variables. The earliest uniform local search algorithm is a tool to evaluate the quality of design. This method has less experiments and can increase the ability of data acquisition. In the local search, two random variables can complete a group of controlled experiments. In the experiment, the experimental variables and control variables are set, and the optimal parameter variables are selected according to the calculated value of the objective function. If the function value of the objective is the best value in the local range, then the two-dimensional space calculation is carried out instead of the original variable. The solution path of this two-dimensional random distribution of search variables is shown in Figure 5.

If the optimal value of a parameter is greater than another parameter, it will replace this parameter for subsequent operations. After introducing Gaussian mutation operator to optimize the search, it can solve the search performance of local close range, every time a mean value of distribution function is generated, and the mean difference matrix is calculated. The result is regarded as the highest numerical coordinate of Gaussian distribution curve. The Gauss variation formula is as follows:

(15) $$\begin{array}{c} \text{stepsize}=\text{normrnd}\left(0,1,N,D\right),\\ X_{i}^{i+1}=X_{i}^{t}+\text{stepsize}⊕X_{i}^{t}. \end{array}$$

**Figure 6:**
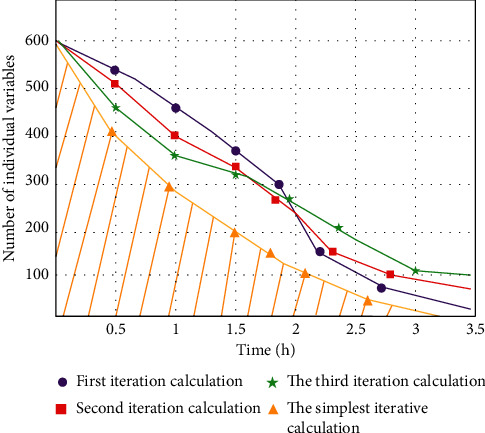
The relationship between the number of individual parameters and the time of optimization model in iterative calculation.

In the process of finding the latest variable value and the global optimal solution, the variable group usually has the phenomenon of numerical error leading to the end of the model calculation. We use Gaussian mutation operator to introduce multiobjective variable group MOPSO algorithm model, which can solve the problem of data diversity and optimize the running speed and performance index of the algorithm. The main process is to add dynamic changes according to real-time mutation and update the number of iterations to get the optimal solution of time flow. The total number of variation individuals will decrease with the increase of optimization model time. The individuals of each generation updating variable will be in the state of decay as a whole and finally get the most simplified iterative individual data. The specific changes are shown in Figure 6.

According to Gauss variogram, the variable calculation expression is as follows:

(16) $$\begin{array}{c} G_{i}\left(t\right)=\frac{1}{\sqrt{2\pi \delta }}\exp\left(-\frac{{\left(x\text{best}-x_{i}^{t}\right)}^{2}}{2\delta^{2}}\right). \end{array}$$

According to the calculation results, we can know that the search path optimized by Gaussian mutation is simpler and faster, which can improve the performance of the whole teaching platform. And the optimized MOPSO algorithm model also increases the speed of feedback information dissemination of teaching platform.

## 4. Analysis on the Application of Gauss Mutation Genetic Algorithm to Optimize Neural Network in Image Painting Art Teaching

### 4.1. Analysis of Research Results of Neural Network Optimized by Genetic Algorithm in Image Painting Art Cloud Platform Teaching

**Figure 7:**
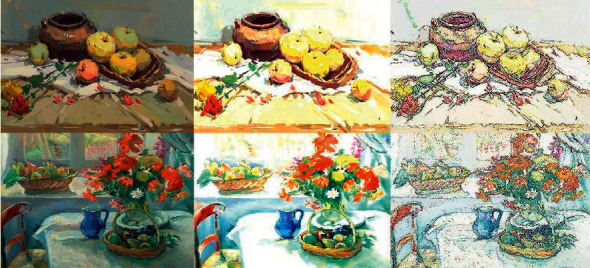
Processing line process chart of drawing image with neural network technology optimized by genetic algorithm.

This paper uses genetic algorithm to optimize the neural network, combined with cloud computing technology to form an online cloud teaching platform. In the image painting art teaching cloud platform, the interaction between various kinds of software and different devices is basically realized, which enables students to independently retrieve knowledge anytime and anywhere and learn image painting art. We use machine learning to make the system training simulate data collection and classify and store the acquired knowledge. Finally, it can be evenly distributed in the platform environment, which improves the performance of the whole server and the feedback response speed. At the same time, we simulate the teaching platform, mainly aiming at the processing mode of the whole system when collecting image information, train the model, and simulate and analyze the image information. The process of processing lines in drawing image data by neural network technology optimized by genetic algorithm is shown in Figure 7.

**Figure 8:**
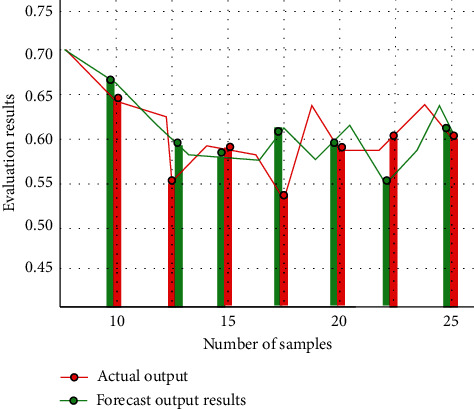
Comparison chart between actual output and predicted output.

Then, we test the teaching quality of the image painting art teaching platform, and the main data index is obtained by using the neural network technology optimized by genetic algorithm. The comparison between the actual output and the predicted output is shown in Figure 8.

According to the experimental results, the prediction results of the neural network model optimized by genetic algorithm are basically consistent with the actual results. Based on the traditional neural network structure, the neural network model optimized by genetic algorithm has higher application value. It can improve the overall performance of the whole teaching application platform and provide effective and scientific evaluation and development direction for image painting art teaching.

### 4.2. Gauss Mutation Optimization Search Algorithm and MOPSO Model of Image Painting Art Teaching Research Results Analysis

**Figure 9:**
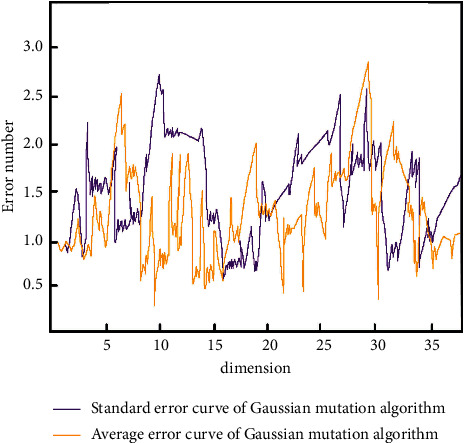
Curves of mean error and standard deviation of Gaussian mutation algorithm in different dimensions.

In order to test the feasibility and performance of Gauss mutation optimization algorithm, we use different problem dimensions to test and evaluate the algorithm. The test function is used to set up multidimensional experiments, so that the Gauss mutation operator can operate independently. The average error and standard deviation of Gaussian mutation algorithm in different dimensions are shown in Figure 9.

According to the curve change in the figure, the average error value of Gaussian mutation algorithm in different dimensions of search performance is less, which can be applied to the establishment of teaching platform mode.

**Figure 10:**
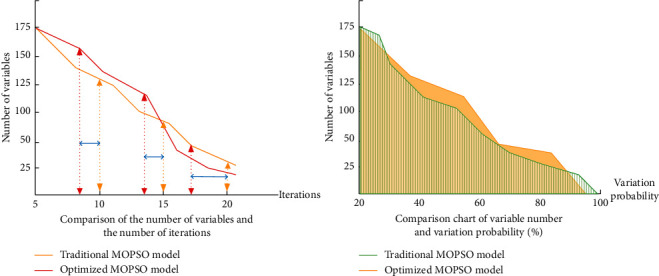
Performance evaluation chart of iteration times and mutation probability affected by the number of variables.

In order to prove the high efficiency of our MOPSO model, this paper evaluates the performance of the traditional model and the optimized model from the number of variables affecting the number of iterations and mutation probability. The index changes are shown in Figure 10.

To sum up, Gauss mutation optimization search algorithm can improve the efficiency of knowledge retrieval data capture in image painting art teaching platform. The overall performance of the teaching model is improved by using Gaussian mutation optimization MOPSO model technology. It can solve the problem of data mixing in the process of multiobjective variables running at the same time and make the image painting art teaching application model to solve the problem of complex data sources and run quickly.

## 5. Conclusion

As people's yearning for a better life and their needs are more and more satisfied, they begin to pay attention to the ability to discover beauty. The art of image painting has gradually become the central knowledge from the art major. There are still many defects in the traditional image painting art teaching, such as the inability to communicate at any time and the scattered distribution of image painting art knowledge. Based on the above situation, this paper proposes a Gauss mutation genetic algorithm to optimize the application of neural network technology in image painting art teaching. The traditional neural network algorithm cannot meet the teaching model to improve performance and operational efficiency. The optimized network structure based on genetic algorithm can reduce the running time and improve the accuracy of the result data. We combine the genetic algorithm optimization neural network with cloud platform to build an application platform for image painting art teaching. The main purpose is to improve the communication and interaction between learners and educators and improve the dispersion of image painting art knowledge. Then, the Gaussian mutation optimization search function is used to improve the process of data information processing. Finally, the Gauss mutation algorithm is used to build the simulation model of image painting art teaching application, and the performance and applicable functions between the traditional MOPSO model and the optimized MOPSO model are compared. The results show that the model optimized by Gauss mutation algorithm has high performance and applicability and can provide effective help for the establishment of image painting art teaching model. However, the model in this paper is computer simulation based on the simulation model, which is somewhat different from the actual data. Therefore, more data research support is needed in future research.

## Data Availability

The data used to support the findings of this study are available from the corresponding author upon request.
